# Supplementary figures and images for: Essential and recurrent roles for hairpin RNAs in silencing de novo sex chromosome conflict in Drosophila simulans
Source: PLoS Biol. 2023 Jun 8;21(6):e3002136. doi: 10.1371/journal.pbio.3002136 (PMC10292708; doi:10.1371/journal.pbio.3002136)

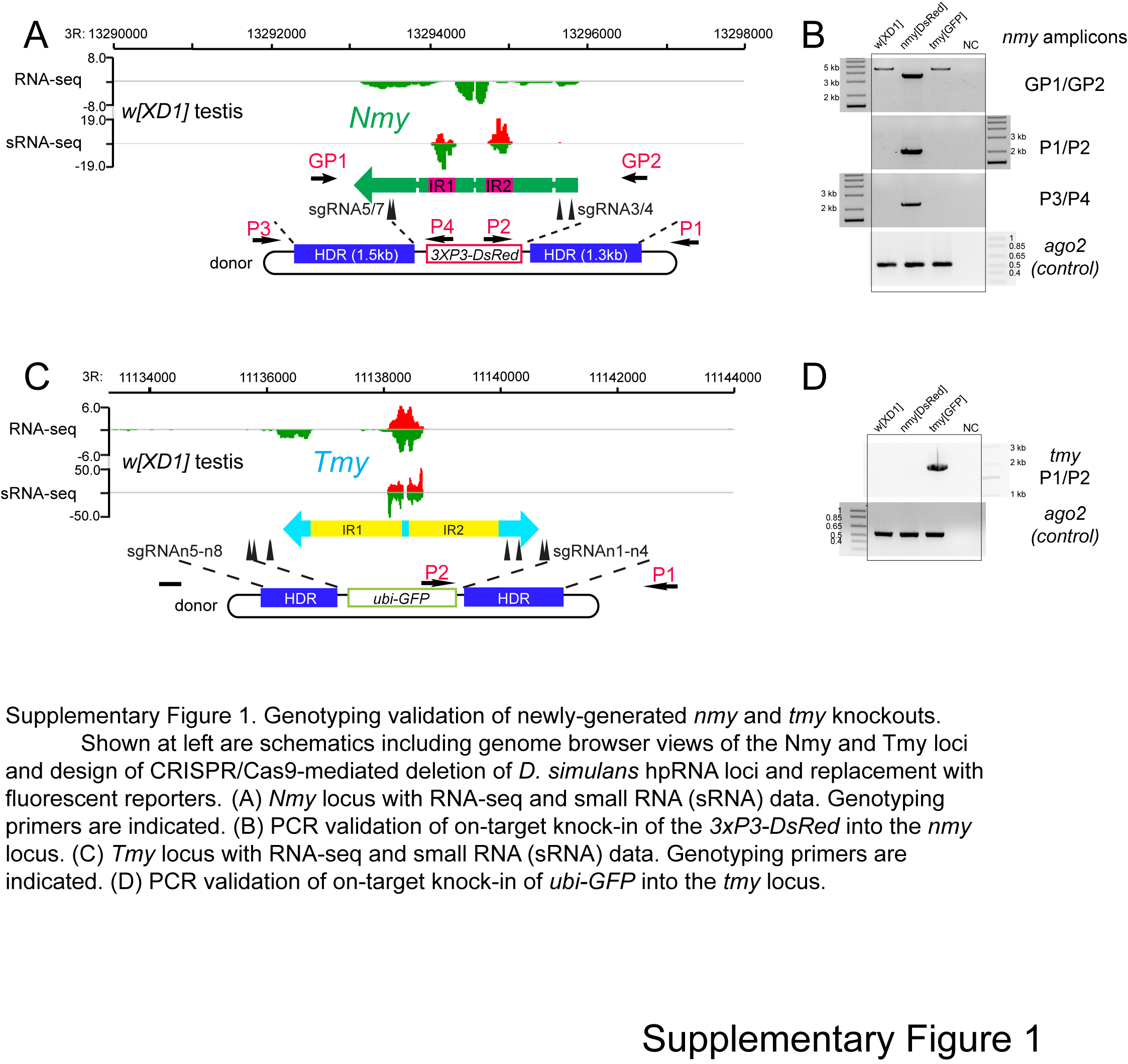

Supplement: S1 Fig — Shown at left are schematics including genome browser views of the Nmy and Tmy loci and design of CRISPR/Cas9-mediated deletion of D. simulans hpRNA loci and replacement with fluorescent reporters. (A) Nmy locus with RNA-seq and small RNA (sRNA) data. Genotyping primers are indicated. (B) PCR validation of on-target knock-in of the 3xP3-DsRed into the nmy locus. (C) Tmy locus with RNA-seq and small RNA (sRNA) data. Genotyping primers are indicated. (D) PCR validation of on-target knock-in of ubi-GFP into the tmy locus. The uncropped genotyping gels are provided in S1 Raw Images. (TIF) [file pbio.3002136.s001.tif]

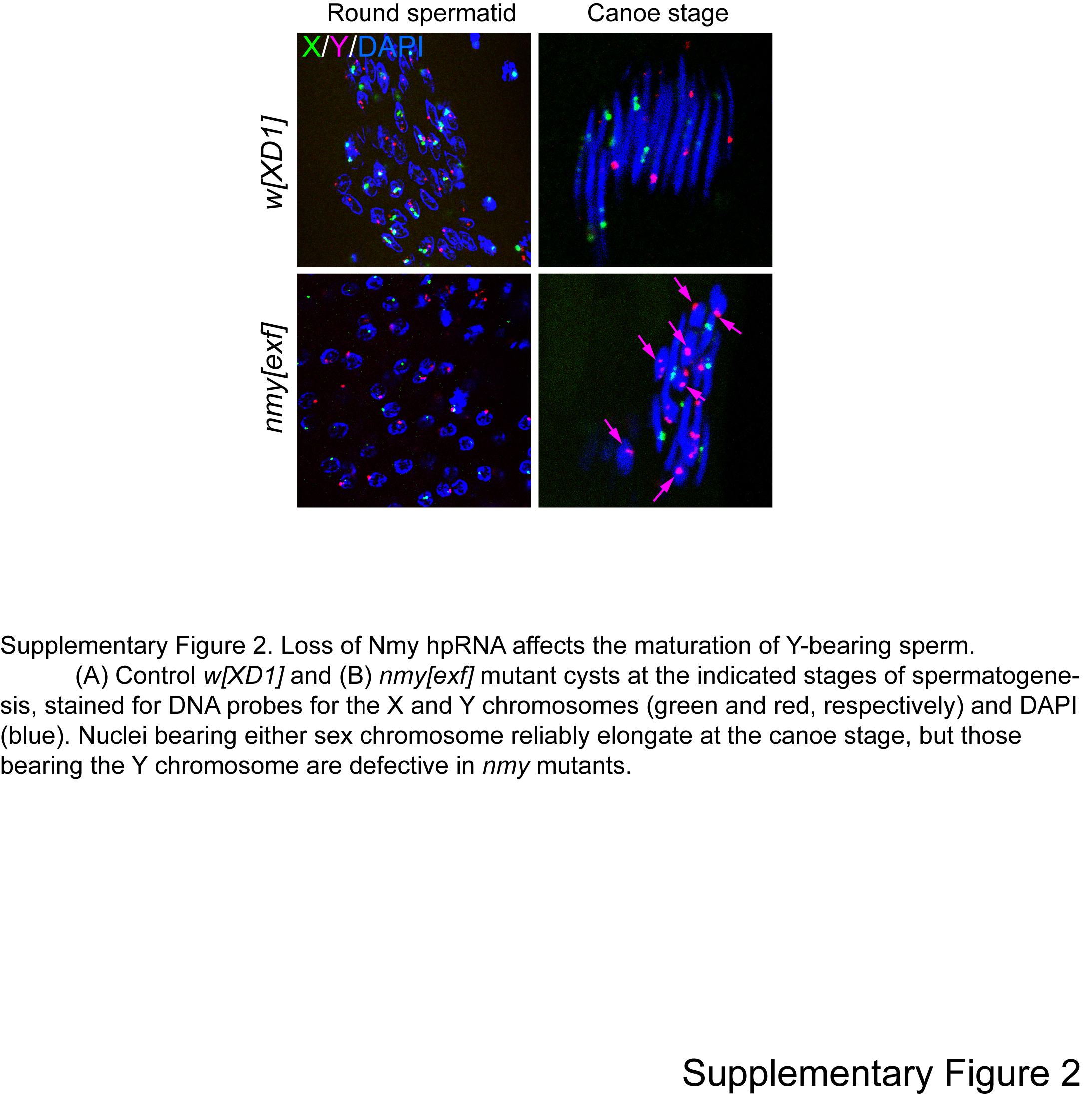

Supplement: S2 Fig — (A) Control w[XD1] and (B) nmy[exf] mutant cysts at the indicated stages of spermatogenesis, stained for DNA probes for the X and Y chromosomes (green and red, respectively) and DAPI (blue). Nuclei bearing either sex chromosome reliably elongate at the canoe stage, but those bearing the Y chromosome are defective in nmy mutants. (TIF) [file pbio.3002136.s002.tif]

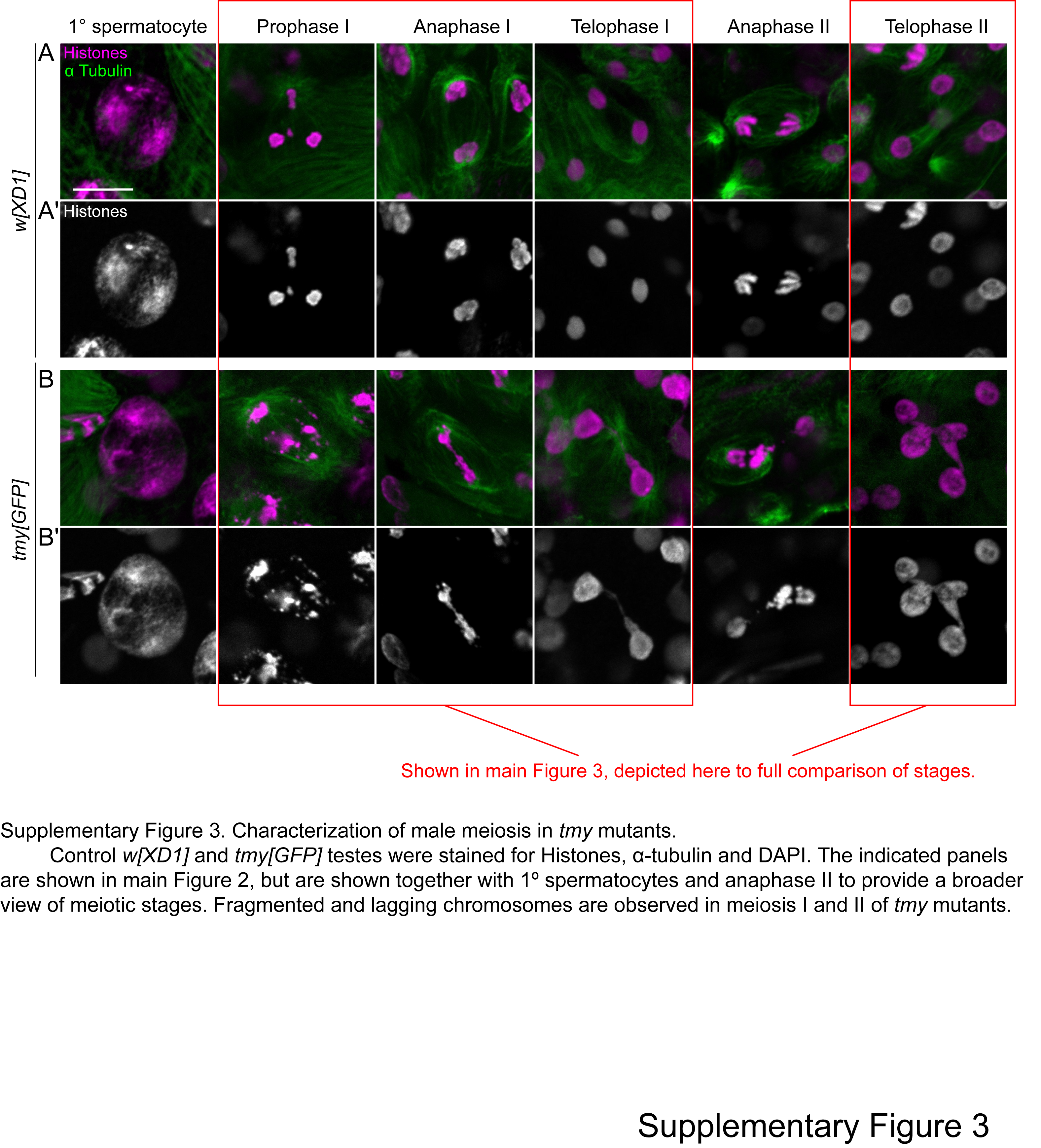

Supplement: S3 Fig — Control w[XD1] and tmy[GFP] testes were stained for Histones, α-tubulin, and DAPI. The indicated panels are shown in main Fig 2, but are shown together with 1° spermatocytes and anaphase II to provide a broader view of meiotic stages. Fragmented and lagging chromosomes are observed in meiosis I and II of tmy mutants. (TIF) [file pbio.3002136.s003.tif]

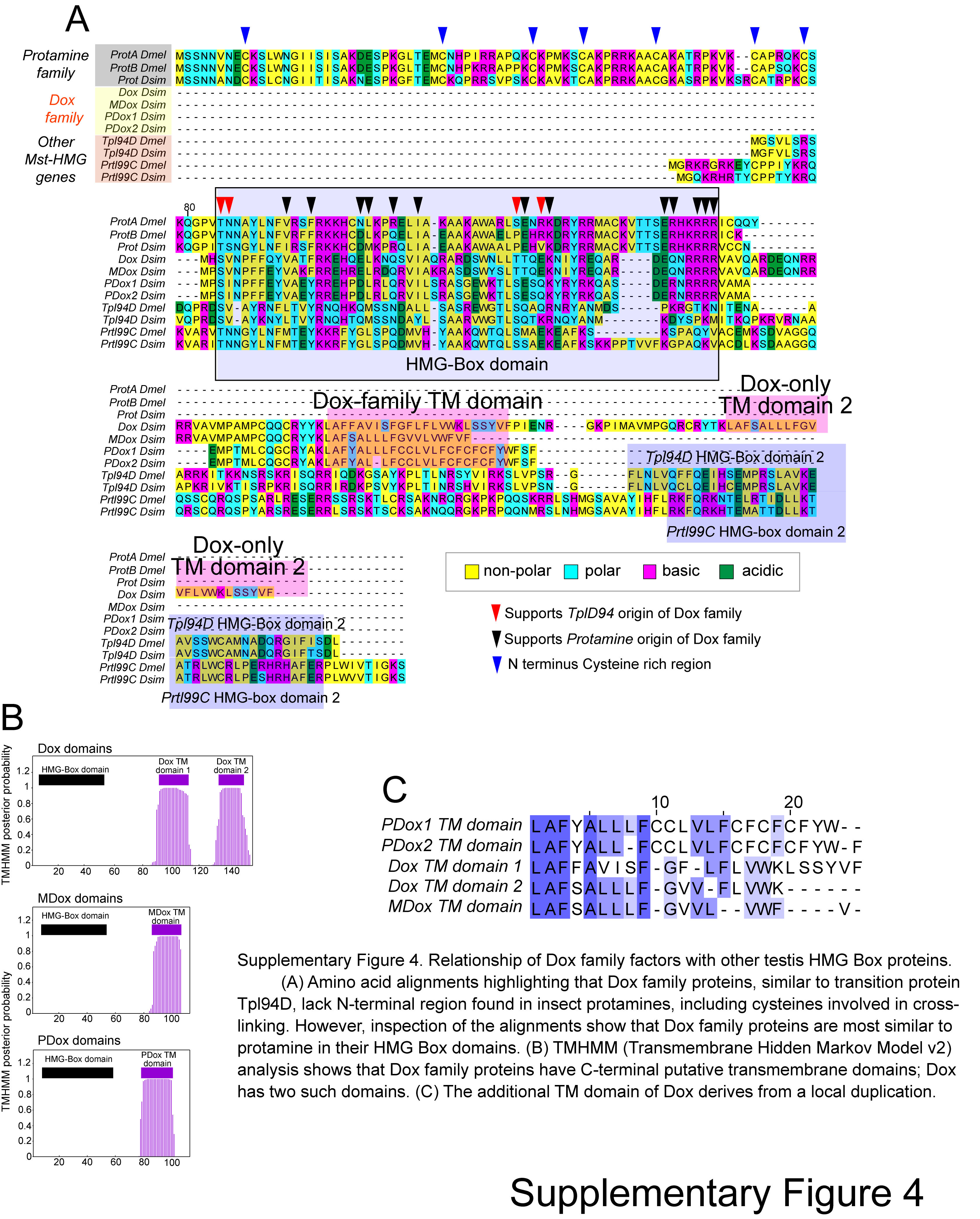

Supplement: S4 Fig — (A) Amino acid alignments highlighting that Dox family proteins, similar to transition protein Tpl94D, lack N-terminal region found in insect protamines, including cysteines involved in crosslinking. However, inspection of the alignments shows that Dox family proteins are most similar to protamine in their HMG Box domains. (B) TMHMM (Transmembrane Hidden Markov Model v2) analysis shows that Dox family proteins have C-terminal putative transmembrane domains; Dox has 2 such domains. (C) The additional TM domain of Dox derives from a local duplication. (TIF) [file pbio.3002136.s004.tif]

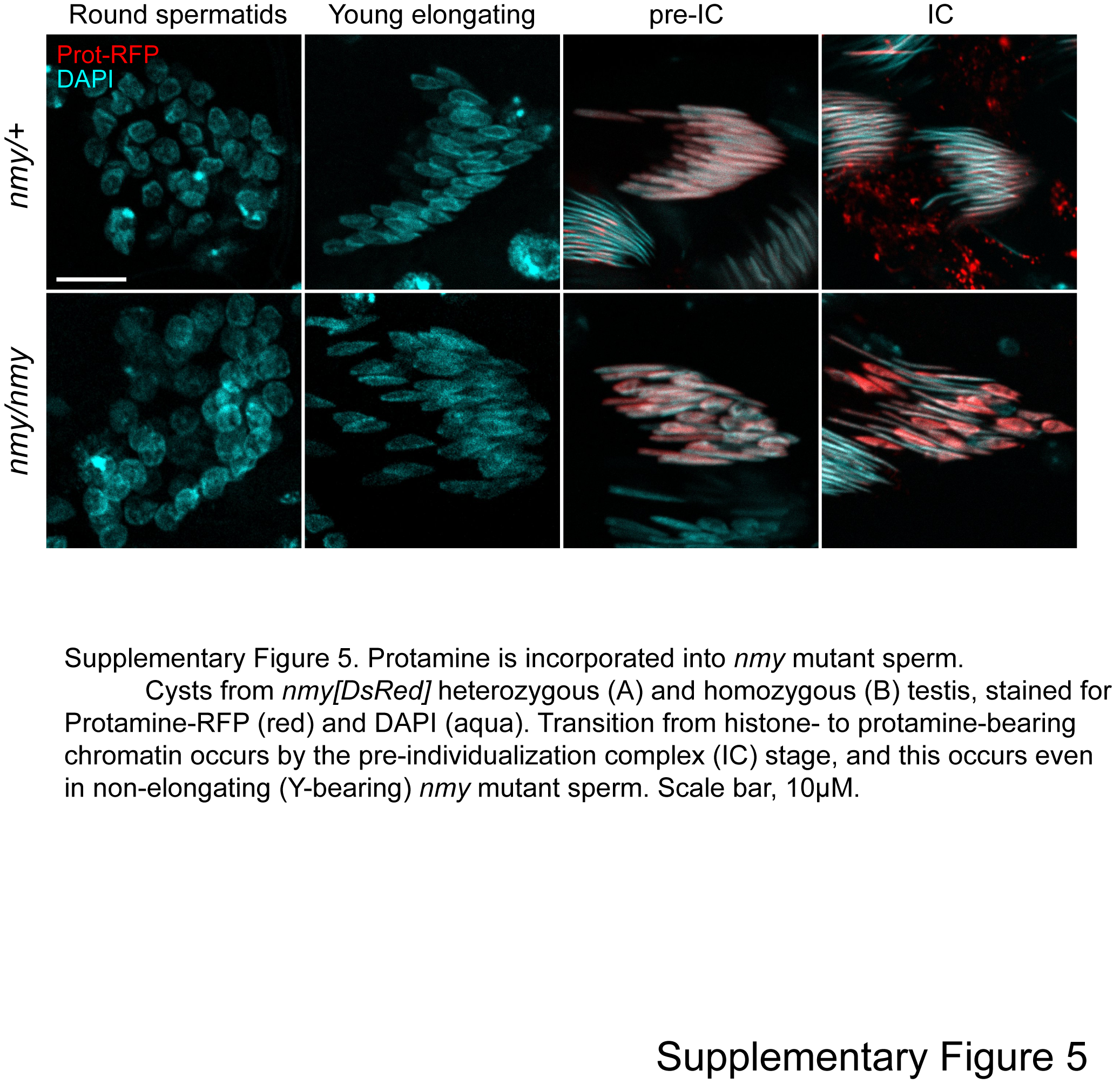

Supplement: S5 Fig — Cysts from nmy[DsRed] heterozygous (A) and homozygous (B) testis, stained for Protamine-RFP (red) and DAPI (aqua). Transition from histone- to protamine-bearing chromatin occurs by the pre-individualization complex (IC) stage, and this occurs even in non-elongating (Y-bearing) nmy mutant sperm. Scale bar, 10 μm. (TIF) [file pbio.3002136.s005.tif]
