# Supplementary material for: Essential and recurrent roles for hairpin RNAs in silencing de novo sex chromosome conflict in Drosophila simulans
Source: PLoS Biol. 2023 Jun 8;21(6):e3002136. doi: 10.1371/journal.pbio.3002136 (PMC10292708; doi:10.1371/journal.pbio.3002136)
Supplement: S1 Raw images — (PDF) [file pbio.3002136.s007.pdf]

Fig 5A

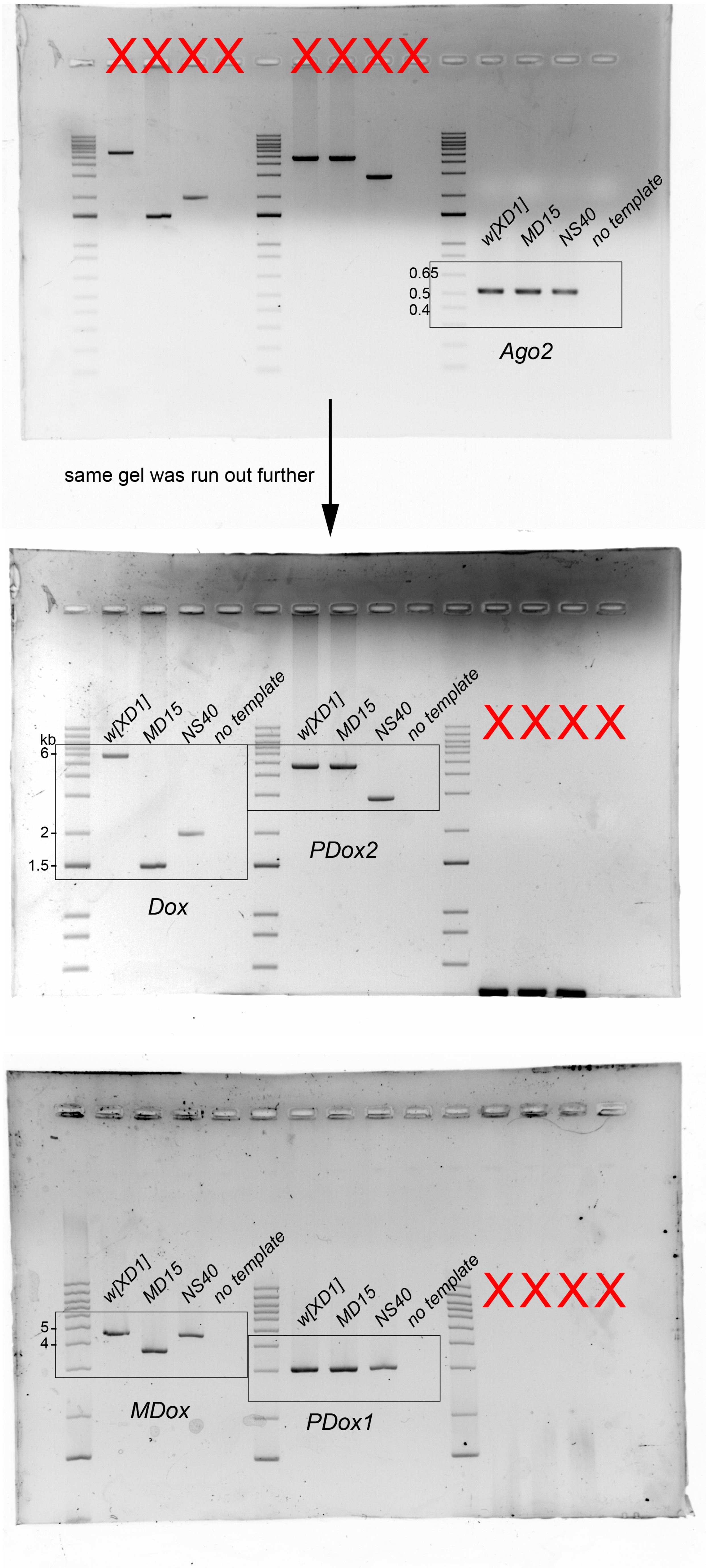

These are the raw uncropped genotyping gels shown in Figure 5A of Vedanayagam and Herbette, et al. The boxed regions are shown in the figure, and were arranged for clarity of comparisons. Red Xs designate gel lanes not used in the figure.

Fig S1B

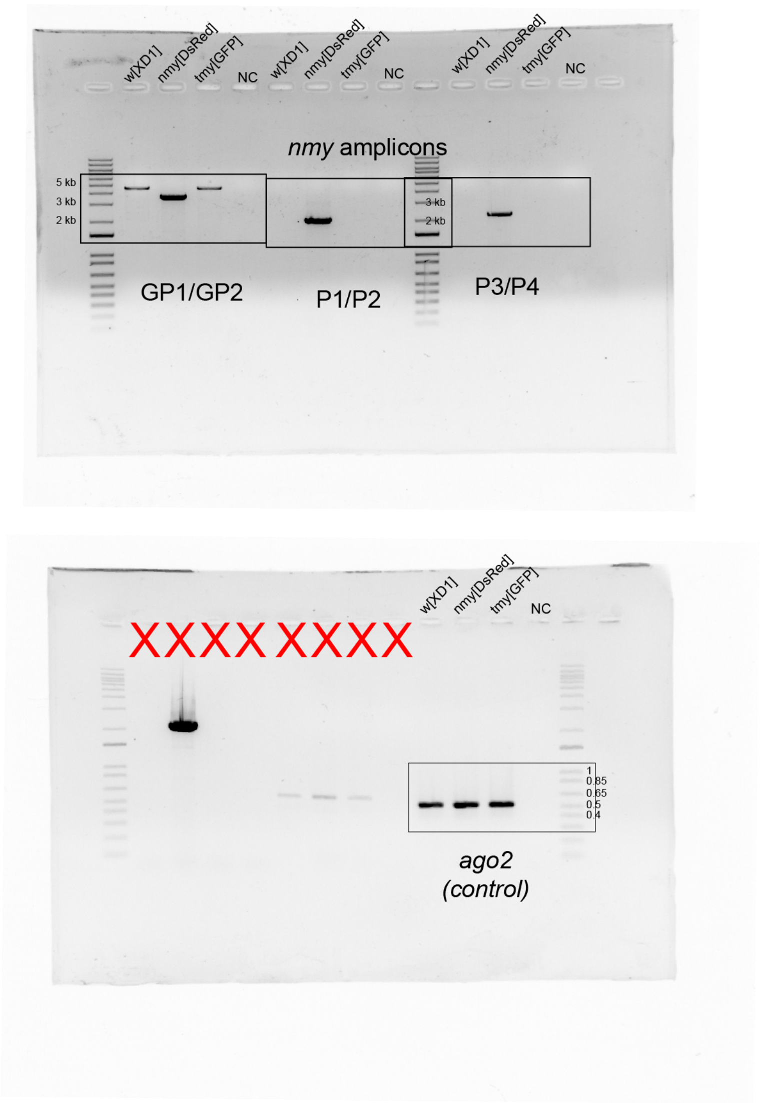

Fig S1D

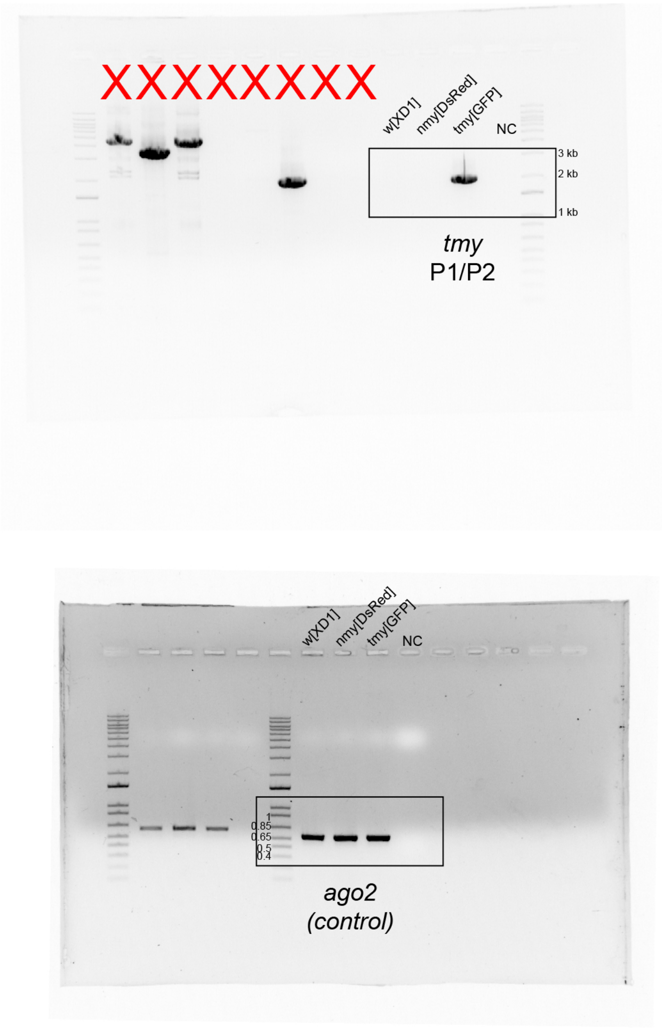

These are the raw uncropped genotyping gels shown in Figure S1B and S1D of Vedanayagam and Herbette, et al. The boxed regions are shown in the figure, and were arranged for clarity of comparisons. Red Xs designate gel lanes not used in the figure.
