# Supplementary material for: Essential and recurrent roles for hairpin RNAs in silencing de novo sex chromosome conflict in Drosophila simulans
Source: PLoS Biol. 2023 Jun 8;21(6):e3002136. doi: 10.1371/journal.pbio.3002136 (PMC10292708; doi:10.1371/journal.pbio.3002136)
Supplement: Attachment — Submitted filename: 2023_0126_Tmy_PDox_reviewer_comments.pdf [file pbio.3002136.s010.pdf]

Dear Dr Lai,

Thank you for your patience while your manuscript "Essential and recurrent roles for endogenous RNAi to silence de novo sex chromosome conflict" went through peer-review at PLOS Biology. Please accept my apologies for the delays that you have experienced during the peer review process. Your manuscript has now been evaluated by the PLOS Biology editors, an Academic Editor with relevant expertise, and by two independent reviewers. Please note that the Academic Editor handling your submission has also provided a review of the manuscript (labelled as 'Academic Editor Comments').

In light of the reviews, which you will find at the end of this email, we are pleased to offer you the opportunity to address the comments from the reviewers and Academic Editor in a revision that we anticipate should not take you very long. We will then assess your revised manuscript and your response to the reviewers' comments with our Academic Editor aiming to avoid further rounds of peer-review, although might need to consult with the reviewers, depending on the nature of the revisions.

I would also be grateful if you could address the following editorial and data-related requests that I provided below (A-F):

(A) We would like to suggest the following minor modification to the title:

“'Essential and recurrent roles for hairpin RNAs in silencing de novo sex chromosome conflict in Drosophila simulans”

We changed the title as suggested.

(B) You may be aware of the PLOS Data Policy, which requires that all data be made available without restriction: http://journals.plos.org/plosbiology/s/data-availability. For more information, please also see this editorial: http://dx.doi.org/10.1371/journal.pbio.1001797

Note that we do not require all raw data. Rather, we ask that all individual quantitative observations that underlie the data summarized in the figures and results of your paper be made available in one of the following forms:

Data are available without restriction and all necessary supplemental tables and data are provided as supplementary files.

- Supplementary files (e.g., excel). Please ensure that all data files are uploaded as 'Supporting Information' and are invariably referred to (in the manuscript, figure legends, and the Description field when uploading your files) using the following format verbatim: S1 Data, S2 Data, etc. Multiple panels of a single or even several figures can be included as multiple sheets in one excel file that is saved using exactly the following convention: S1_Data.xlsx (using an underscore).

- Deposition in a publicly available repository. Please also provide the accession code or a reviewer link so that we may view your data before publication.

Accession code to data included in this paper is provided in the manuscript under the data access section.

Regardless of the method selected, please ensure that you provide the individual numerical values that underlie the summary data displayed in the following figure panels as they are essential for readers to assess your analysis and to reproduce it:

Figure 1E, 1I-J, 3A-C, 5B-D

The designated raw data for these plots are provided as supplementary files.

NOTE: the numerical data provided should include all replicates AND the way in which the plotted mean and errors were derived (it should not present only the mean/average values).

(C) We ask that you please deposit the RNA-seq and sRNA-seq data presented in Figure 1B and 1F in a public repository, such as the GEO. Please ensure that the data is made publicly available and the accession number(s) are provided in the Data Availability Statement in the online submission form upon re-submission.

The data for RNA-seq and sRNA-seq data presented in Figure 1B and 1E are deposited in the GEO database under the accession number GSE185361 from a prior publication (Vedanayagam et al. 2021 NEE)

*(D) Please also ensure that each of the relevant figure legends in your manuscript include information on *WHERE THE UNDERLYING DATA CAN BE FOUND*, and ensure your supplemental data file/s has a legend.*

(E) We require the original, uncropped and minimally adjusted images supporting all blot and gel results reported in the following Figures:

Figure 1D, 1H, 5A

Original uncropped gel images supporting all the gel images shown in the paper are included in the revised version of the manuscript.

We will require these files before a manuscript can be accepted so please prepare and upload them now. Please carefully read our guidelines for how to prepare and upload this data: https://journals.plos.org/plosbiology/s/figures#loc-blot-and-gel-reporting-requirements

(F) Please ensure that your Data Statement in the submission system accurately describes where your data can be found and is in final format, as it will be published as written there.

As you address these items, please take this last chance to review your reference list to ensure that it is complete and correct. If you have cited papers that have been retracted, please include the rationale for doing so in the manuscript text, or remove these references and replace them with relevant current references. Any changes to the reference list should be mentioned in the cover letter that accompanies your revised manuscript.

We expect to receive your revised manuscript within 1 month. Please email us

(plosbiology@plos.org) if you have any questions or concerns, or would like to request an extension.

At this stage, your manuscript remains formally under active consideration at our journal; please notify us by email if you do not intend to submit a revision so that we withdraw the manuscript.

****IMPORTANT - SUBMITTING YOUR REVISION*****

Your revisions should address the specific points made by each reviewer. Please submit the following files along with your revised manuscript:

1. A 'Response to Reviewers' file - this should detail your responses to the editorial requests, present a point-by-point response to all of the reviewers' comments, and indicate the changes made to the manuscript.
**NOTE: In your point-by-point response to the reviewers, please provide the full context of each review. Do not selectively quote paragraphs or sentences to reply to. The entire set of reviewer comments should be present in full and each specific point should be responded to individually.*
You should also cite any additional relevant literature that has been published since the original submission and mention any additional citations in your response.

2. In addition to a clean copy of the manuscript, please also upload a 'track-changes' version of your manuscript that specifies the edits made. This should be uploaded as a "Revised Article with Changes Highlighted " file type.

Resubmission Checklist
When you are ready to resubmit your revised manuscript, please refer to this resubmission checklist: https://plos.io/Biology_Checklist

To submit a revised version of your manuscript, please go to https://www.editorialmanager.com/pbiology/ and log in as an Author. Click the link labelled 'Submissions Needing Revision' where you will find your submission record.

Please make sure to read the following important policies and guidelines while preparing your revision:

Published Peer Review
Please note while forming your response, if your article is accepted, you may have the opportunity to make the peer review history publicly available. The record will include editor decision letters (with reviews) and your responses to reviewer comments. If eligible, we will contact you to opt in or out. Please see here for more details:
https://blogs.plos.org/plos/2019/05/plos-journals-now-open-for-published-peer-review/

PLOS Data Policy
Please note that as a condition of publication PLOS' data policy (http://journals.plos.org/plosbiology/s/data-availability) requires that you make available all data used to draw the conclusions arrived at in your manuscript. If you have not already done so, you must include any data used in your manuscript either in appropriate repositories, within the body of the manuscript, or as supporting information (N.B. this includes any numerical values that were used to generate graphs, histograms etc.). For an example see here: http://www.plosbiology.org/article/info%3Adoi%2F10.1371%2Fjournal.pbio.1001908#s5

Blot and Gel Data Policy
We require the original, uncropped and minimally adjusted images supporting all blot and gel results reported in an article's figures or Supporting Information files. We will require these files before a manuscript can be accepted so please prepare them now, if you have not already uploaded them. Please carefully read our guidelines for how to prepare and upload this data: https://journals.plos.org/plosbiology/s/figures#loc-blot-and-gel-reporting-requirements

Protocols deposition
To enhance the reproducibility of your results, we recommend that if applicable you deposit your laboratory protocols in protocols.io, where a protocol can be assigned its own identifier (DOI) such that it can be cited independently in the future. Additionally, PLOS ONE offers an option for publishing peer-reviewed Lab Protocol articles, which describe protocols hosted on protocols.io. Read more information on sharing protocols at https://plos.org/protocols?utm_medium=editorial-email&utm_source=authorletters&utm_campaign=protocols

Thank you again for your submission to our journal. We hope that our editorial process has been constructive thus far, and we welcome your feedback at any time. Please don't hesitate to contact us if you have any questions or comments.

Sincerely,

Richard

Richard Hodge, PhD
Associate Editor, PLOS Biology
rhodge@plos.org

--

REVIEWS:

Reviewer #1: This paper addresses the recent observation that a sex ratio drive gene (Dox) and its suppressor (Nmy) have been recurrently duplicated in the D. simulans genome, suggesting ongoing cycles of meiotic drive and suppression. The authors use reverse genetics, together with cytology and epistasis to reveal that a novel trio two Dox paralogs (ParaDox) and one suppressor (hpTmy) also impact male fertility, with ParaDox expression disrupting spermatogenesis if not controlled by Tmy. They further show for the first time that Dox family members code for proteins that are produced only in the absence of their cognate repressors, which are expressed specifically in developing sperm. This findings are very novel in suggesting for the first time how spermatotoxic proteins and repressor pairs can arise concurrently through gene duplications.

I did not review the original submission of this paper, nor did I have access to the authors response to review. I found this version of the manuscript well written, although somewhat lengthy in parts. The experiments are thorough and thoughtfully laid out. The findings are novel and I believe of interest to a general audience. I truly enjoyed reading this paper and congratulate the authors and their excellent science.

We thank the reviewer for their enthusiasm and positive assessment of our study. This study was not previously reviewed, and thus there is no prior response to review.

My only significant concerns about the paper are in the discussion. First, I find there is a bit of shoe-horning when it comes to the results with ParaDox1/2. The phenotype resulting from misexpression of these two proteins is complete male sterility, which differ from that of Dox and is not necessarily indicative of meiotic drive. This observation is REALLY interesting and puzzling, since obviously ParaDox1/2 paralog would be highly deleterious and never fix if it arose on a Tmy mutant background. How do the authors envision ParaDox1/2 originally spread? Perhaps is showed drive, but a X-linked suppressor was secondarily lost after Tmy emerged? Perhaps it emerged after Tmy and persisted because it was neutral? The authors must have thought about this and I would be interested for them to better interpret this result in the discussion. As it currently stands, making a blanket claim that ParaDox1/2 and Tmy represent just another example of a SR distorter and its suppressor seems like a stretch.

We thank the reviewer for highlighting the puzzling inferred activity of *ParaDox1/2* genes to induce sterility, especially as we infer that they harbor meiotic drive activity and are spreading in the *simulans* clade. On one hand, the *Tmy* mutant males are sterile in the *w[XD1]* background. Our genetic interaction experiments, on the other hand, highlighted a role for *Tmy* in sex-ratio control. Thus, it is possible that *PDox* arose as a sex-ratio driver and spread in the population similar to contemporary *Dox* distorter. One line of evidence that supports this claim is the observation from prior genetic studies where *Tmy* was identified as a suppressor of sex-ratio distortion. In the genetic background where *Tmy* was originally characterized, *tmy* introgression segments were also linked to semi-sterility/sterility phenotype. Thus, it is reasonable to speculate that the genetic background alters the strength of the drive phenotype ranging from sex-ratio distortion to semi-sterility/full sterility.

Based on this prediction, we observed complete rescue of sterility with X chromosome derived from wild isolate NS40 bearing natural deletion at the *PDox2* locus. However, if genetic background dictates the strength of the drive phenotype, we may expect to observe sex-ratio distortion in other wild isolates (similar to sex-ratio distortion observed by Tao et al. 2001 in the *simB* genetic background). To test this prediction, in the future we consider to examine further wild X chromosomes to see if any reveal sex-ratio distortion in the presence of tmy-KO, akin to what was observed by Tao *et al*. 2001. Such tests are beyond the scope of the current manuscript. However, we revised the text to explain our results in the historical context of genetic identification of the *Tmy* locus, and our functional characterization of PDox loci in the *w[XD1]* genetic background where sterility is observed upon knockout of the *Tmy* hpRNA.

My second more minor concern is the discussion the histone to protamine transition. On pages 18-19 it is staged that "…one emerging principle is that the histone-to-protamine transition is a critically sensitive stage of spermatogenesis, and the molecules responsible for effecting this process may frequently be co-opted to during the evolution of meiotic drive in the male germline." The data in the paper show that the Dox paralogs do not impact the incorporation of protamines although they are produced in sperm at this time in the absence of repression. Thus it seems to me that while this stage may be "critically sensitive" the data in this paper do not support Dox as effectors of this transition.

We agree with the referee " *the data in this paper do not support Dox as effectors of this transition"*. However the discussion statement they refer to: "*However, one emerging principle is that the histone-to-protamine transition is a critically sensitive stage of spermatogenesis, and the molecules responsible for effecting this process may frequently be co-opted to during the evolution of meiotic drive in the male germline*." is made in reference to the collected literature

from literature, including the role of protamine in Segregation Distorter, and positive selection on protamine/SNBP genes and copies across Drosophila species. We acknowledge that our work does not provide evidence that newly emerged meiotic drive factors in the Dox family, derived from protamines, can cytologically inhibit protamine incorporation. We have revised the text description to be very explicit about the limitations of the tagged transgenes experiments, with respect to impact on chromatin organization.

However, it is also the case that immunostaining is not a very sensitive technique and additional genomic experiments await in the future. On the other hand, where we discuss this in the discussion, it seems appropriate given the collected literature on protamines and their derivatives as agents of sex chromosome conflict and/or meiotic drive.

A few minor comments:

Nmy and Tmy should always be in italics since they do not code for proteins.

All instances of *Nmy* and *Tmy* in the manuscript are italicized in the revised manuscript.

One page 13 I am not comfortable with the following sentence "…we do find that protamine incorporation fails completely in ago2 mutant testis (Figure 4P), indicating that net derepression of endo-siRNA targets blocks the histone-to-protamine transition."

The conclusion that can be drawn from this experiment is that ago2 is required for protamine incorporation. Whether that requirement arises from the need to repress siRNA targets is a reasonable hypothesis that would need to be tested before a conclusion could be drawn.

We agree with the reviewer, and modified this to state that these events are simply "associated" (eg we cannot distinguish a causal relationship at this point).

Reviewer #2: In this manuscript, Vedanayagam and colleagues explore in detail the biology of two hairpin RNA (hpRNA) loci that have been previously implicated as suppressors of X-linked meiotic drive elements in hybrids between two fly species D. mauritania and D. simulans. In particular, the authors investigated whether these hpRNAs are redundant in their repressive roles (shared vs unique targets) and the consequences of their loss at the cellular and molecular level in D. simulans. I think this is a very important study that helps us better understand, with unprecedented molecular detail, how genetic conflict drives genomic evolution. Overall, I found that the manuscript is well written, the logic of the experiments is clearly laid out, and the results are discussed accordingly.

We thank the reviewer for the positive comments on the manuscript.

Major points

One key contribution of this study is that it allows for a fair comparison between Tmy and Nmy hpRNAs. The authors took great care to generate new mutant alleles to exclude confounding effects due to the random effects caused by introgressions, mutations, and genetic modifiers segregating in populations.

I found the experiments described in figure 4 particularly exciting. Although not the endogenous Dox loci, the visualization of Dox and Dox-like proteins from tagged transgenes in hpRNA

mutant background will definitely help characterize the biology of this mysterious protein family. Likewise, I found the rescue of hpRNA mutants using naturally occurring Dox mutant genetic variants quite elegant. I'm sorry if I missed this, but did the authors also characterize the hpRNAs loci in these wild lines (MD15 and NS40). Is it possible that in the absence of Dox and related genes, hpRNAs may have accumulated also deleterious mutations in the parental lines? Thus could help understand the evolutionary dynamics of such systems in the wild.

Our current crossing scheme introduces the *nmy* and *tmy* mutant alleles in the wild isolate background, while retaining the X from the wild isolate. The suggestion made by reviewer is interesting to examine the evolutionary dynamics of drivers and suppressors in the wild lines, and we aim to pursue this in future by characterizing these wild chromosomes in further detail. We acknowledge that they differ from w[XD1] along their lengths, so it will be important to gain more precise evidence that specific loss of Dox family genes mitigates the deletion of hpRNAs.

We predict that once the drivers are lost, eventually the hpRNA loci may either accumulate deleterious mutations or lost from the parental lines, and our future studies in this directions will help characterize the dynamics of these systems at the population level.

Regarding the Dox gene family. One criticism that I have is that the language is inconsistent and sometimes confusing. For instance, "Dox genes are not replicas of canonical protamines" (page 12), "(they) are simply protamine mimics" (page 12), "Dox family encodes illegitimate protamine copies" (figure 4 legend). To me, the term mimicry would imply convergent evolution, and I didn't see any evidence for that. I would also hesitate to call the genes "illegitimate" copies. What is legitimate for a cell after all? If the authors think these Dox families evolved by gene duplication and diversification, then they are simply "highly divergent paralogs". Related to this point, is it possible that Dox and Dox-like proteins are simply "toxins" as the ones observed in prokaryotic and eukaryotic selfish toxin-antitoxin systems? (In this case a toxin is defined as a protein that is poisonous above a certain threshold of expression but otherwise dispensable for the organism)

We rephrased the description of *Dox* family genes to reflect their origins as highly divergent derivatives of *Protamine*, we removed the "illegitimate" and "mimic" qualifiers, since as acknowledged, we don't yet fully know the biochemical functions of Dox family proteins. However, we do feel that "illegitimate" is appropriate, since they do not clearly localize as with bona fide Protamine and transition proteins. In any case, we removed these adjectives.

When tmy is mutated, there is no change in male/female ratio but males are sterile. How does this observation fit in the model that hpRNAs evolved to suppress meiotic drive of sex chromosomes? From this data, I would conclude that tmy is suppressing is not a "meiotic driver" (defined as a genetic element that biases meioses to increase its frequency in populations). Please correct me if I'm wrong, but theoretically, if I mix two populations, one "wt" and the other one carrying the dox locus (both lacking hpRNA suppressors), then the dox+ allele would be negatively selected and lost from the population. Thus, I don't think it would qualify as a meiotic driver. Thus, tmy simply seems to suppress a gene that is toxic when expressed in males during meiosis.

We thank the reviewer for highlighting the puzzling inferred activity of *ParaDox1/2* genes to induce sterility, especially as we infer that they harbor meiotic drive activity and are spreading in the *simulans* clade. On one hand, the *Tmy* mutant males are sterile in the *w[XD1]* background. Our genetic interaction experiments, on the other hand, highlighted a role for *Tmy* in sex-ratio

control. Thus, it is possible that *PDox* arose as a sex-ratio driver and spread in the population similar to contemporary *Dox* distorter. One line of evidence that supports this claim is the observation from prior genetic studies where *Tmy* was identified as a suppressor of sex-ratio distortion. In the genetic background where *Tmy* was originally characterized, *tmy* introgression segments were also linked to semi-sterility/sterility phenotype. Thus, it is reasonable to speculate that the genetic background alters the strength of the drive phenotype ranging from sex-ratio distortion to semi-sterility/full sterility.

Based on this prediction, we observed complete rescue of sterility with X chromosome derived from wild isolate NS40 bearing natural deletion at the *PDox2* locus. However, if genetic background dictates the strength of the drive phenotype, we may expect to observe sex-ratio distortion in other wild isolates (similar to sex-ratio distortion observed by Tao et al. 2001 in the *simB* genetic background). To test this prediction, in the future we consider to examine further wild X chromosomes to see if any reveal sex-ratio distortion in the presence of tmy-KO, akin to what was observed by Tao *et al*. 2001. Such tests are beyond the scope of the current manuscript. However, we revised the text to explain our results in the historical context of genetic identification of the *Tmy* locus, and our functional characterization of PDox loci in the *w[XD1]* genetic background where sterility is observed upon knockout of the *Tmy* hpRNA.

Related to the previous point. Is there any reason to believe that hpRNAs suppress exclusively or mainly meiotic drive elements found in the X chromosome? As pointed out by the authors, such elements are easier to spot when located in sex chromosomes because they cause deviations in the expected 1:1 sex ratio. But is there any empirical data indicating that they preferentially evolve in sex chromosomes?

In D. melanogaster, where we studied this topic originally, there is not a very substantial bias for hpRNA targets to target X chromosome genes (although there are some). Also, as noted in the text and in our previous paper, there is neither evidence for a strong sex-biasing selfish meiotic drive system after >100 years of D. melanogaster genetics, nor do core RNAi mutants exhibit sex ratio bias in D. melanogaster.

What this study demonstrates, and extends our previous work (Lin Dev Cell 2018), is that there are evolutionarily recurrent and evolutionarily nascent examples of sex chromosome conflict that absolutely require individual hpRNA loci for suppression. All of the D. melanogaster hpRNAs, while "young" on the absolute scale, are relatively "old" for hpRNAs, since Nmy and Tmy are much younger than all D. melanogaster hpRNAs (which are also retained in D. simulans). Our speculation is that such extremely young hpRNAs may serve functional roles of greater phenotypic importance than older ones, and sex chromosome conflict could be one of those critical settings. Clearly there must be other biological rationale for evolutionarily short-lived regulation of autosomal genes by hpRNAs, but these require further study to comprehend.

Minor points

It would be good to have a bit more color consistency in Figures. For example, in Figure 1,why would panels 1E and 1I use different colors? They are both depicting the same measurement (male fertility). Or are they supposed to match the colors assigned to Tmy (light blue) and Nmy (green)? Even though the color in 1E is not really the same as 1B. In later figures, the color code of Tmy and Nmy changes once again (see Fig 3E, for example, both are different shades

of blue). I would encourage authors to make this a bit easier for reades who are not familiar with this system (it's hard to keep track of tmy/nmy and dox/Mdox/Pdox, etc)

We thank the reviewer for raising this point. We have now changed the coloring scheme for *Nmy* and *Tmy* genotypes consistent in all figures in the manuscript.

Typo in Figure 6. "Theis existence"

We corrected this typo in Figure 6. Thank you for your close reading!

The color of boxplots in Figure 3C (white versus gray) is really hard to see.

Figure 3C is now Figure 4C in the revised manuscript and we have made the panel larger and have highlighted the boxplots in black versus red to show the contrast.

ACADEMIC EDITOR COMMENTS

Vedanayagam et al explore the functions of the Dox family of X-linked meiotic drivers and their suppressors in D. simulans. Tmy and Nmy were both previously implicated as autosomal suppressors of the meiotic drivers. This work provides strong experimental support that both genes are drive suppressors and provides additional insight about both genes via analyzing deletion mutants. They show that nmy homozygous mutant males are fertile and show strong drive of an X chromosome containing the Dox and MDox drive genes, but not an X chromosome without those genes. The authors find that tmy homozygous mutant males are sterile in a background that contains Dox and PDox genes, but not in a background lacking those genes. In addition, the authors find X chromosome drive in tmy/+ nmy/nmy males, but not +/+ nmy/nmy males raised at 25degrees, further supporting the role of Tmy as a drive suppressor. The authors also characterized testis morphology of both mutants and showed the localization of tagged Dox and PDox2 proteins in testes. The work makes significant progress toward understanding Dox family drivers and should guide and facilitate future work characterizing the mechanism(s) of Dox family drive. This is an interesting and important contribution to the meiotic drive and siRNA fields. The claims are mostly sufficiently supported. I point out a few concerns and suggestions for improving the work below.

Major points:
The abstract claims that the localization of the PDox protein indicates that it has a chromosomal function. I was not convinced the data supported this claim and suggest the abstract is reworded. My first concern is that it isn't clear to me how the authors determined that the fusion proteins were function. My second concern is that the PDox signal was not exclusively on chromosomes, as the abstract set me up to expect. Given the homology between Dox and PDox and that they both cause drive, one could argue that the cytoplasmic co-localization shared by the two proteins in meiosis is what is actually important for drive. Also, the tmy/tmy mutants seem to have a significant defect well before chromosome segregation.

Figures 2, 4 and 5. I found much of the cytology quite data hard to see. Some of that was because of the choice of colors and presenting merged images. For example, it is hard to see red and blue on the black background. I suggest showing greyscale individual panels and then show the colors in merged images. That will also make the data accessible to color blind readers.

In addition to the color thing mentioned above, I suggest adding cartoon explain key parts of the

gonad as a new Figure 1A. I also suggest more arrows and such pointing out key features in the data. For example, I can see the seminal vesicle in WT in 1A, but I was not totally sure which little nubs were the seminal vesicle in the mutants.

We added a new figure (figure 2) describing a cartoon to explain the key features of Drosophila spermatogenesis. We decided it made sense to insert after Figure 1, but directly before the first relevant figure (Fig 3) describing testis cytology.

Minor Points:
I was often confused by the genotypes assayed and suggest being more explicit when writing the genotypes in the figures and text (i.e. present homozygotes as 'nmy/nmy' or 'nmy homozygotes'). For example, on page 6, paragraph 2 says that all the trans heterozygotes were fertile then later said 'all tmy[GFP] genotypes were completely male sterile.' The latter half of the sentence sounds like tmy[GFP] heterozygotes are sterile too.

We've revised the manuscript to use a consistent notation to describe the mutant genotype.

I suggest introducing the ubi-GFP and 3xp3-DsRed markers to be more accessible to non-fly researchers.

Figure 1G is hard to see the red on black. It would likely be easier to see if it was switched to greyscale.

We changed this figure panel to grayscale.

Figure 1H: I am confused by this gel. 4.5 and 3.2kb bands should not be hard to separate on a gel, but the bands are not distinguishable, and the ladder does not seem to be on the same gel.

The figure panel 1H is now moved to supplementary figure 1 and we've replaced the figure with a new gel image that shows 4.5 and 3.2 kb products legibly.

Is the nmy allele in supplemental figure 1 the same DsRed disruption? The label is different.

We corrected this and in the revised manuscript, the previous supplementary figure 1 is now supplementary figure 2.

At the bottom of page 10 it says both hpRNAs are dominant. This should be reworded to say wild-type Tmy is dominant given that data from nmy heterozygotes are not presented.

We corrected this in the revised manuscript.

Figure 3: I think the genotype label in 3C is incorrect. The drive observed in the tmy/+, nmy/nmy males at high temp is quite cool. To fully understand that result, I want to know if Tmy is not completely dominant at 25degrees in the background used in this study. In other words, i was not convinced the phenotype in the tmy/+, nmy/nmy was due to Dox/MDox, not PDox. Also, it is important to know if the drive phenotypes correlate with the levels of Dox/MDox/PDox expression at those temperatures.

It's an interesting experiment but we are technically unable to address this at present. The isolation of sufficient testis for qPCR experiments is a very demanding exercise requiring heavy dissection and absolute purity of genotypes (no "wrong" animals selected). Unfortunately we no longer have an experimentalist in the lab to carry out this work and are desperately seeking one, but we are not in the position to carry this out at present. However, we do not think that this

result changes our major conclusions on Tmy genetics, the first ever reported using specific alleles. Many future experiments can be done and will be done.

In Figure 3 and figure 5, the 'n' for some of the experiments is unclear (e.g. number of dots on plots is unclear). It would be good to mention those numbers in the figure legend or on the plots.

We included “n” for all the experiments shown in Figures 3 and 5 in the revised manuscript figures.

In Figure 4, Panels E and J are not labeled or explicitly described in the legend. I was not sure what part of the gonad that was or what the white boxes are indicating. In M, the text says protamine should accumulate in the seminal vesicle but I cant see that in this image. Part of the problem is likely the colors, but the SV is also cut off in the image.

In Figure 5, it is not clear what those PCR rxns are. I suggest a cartoon like that used in 1F to illustrate the locations of the oligos.

We added a new cartoon to explain the *Dox* family genotyping PCR in figure 5.

For PLOS Biology's broad audience, the discussion should be broadened to include sex chromosome drivers found outside of insects.

Sex chromosome conflicts occur broadly throughout sexually reproducing organisms. However, to our knowledge, D. simulans is the first system that directly connects this to small RNA control. We feel the discussion is sufficiently extensive to address key and contemporary concepts in the roles of endogenous RNAi and mechanisms of male meiotic drive. We are open to editorial discussion on this, but this may be more appropriate for a review article than our current study, which is pretty long as it is.
