# Supplementary material for: Essential and recurrent roles for hairpin RNAs in silencing de novo sex chromosome conflict in Drosophila simulans
Source: PLoS Biol. 2023 Jun 8;21(6):e3002136. doi: 10.1371/journal.pbio.3002136 (PMC10292708; doi:10.1371/journal.pbio.3002136)
Supplement: Attachment — Submitted filename: 2023_0401_Tmy_PDox_2ndrev_responses.pdf [file pbio.3002136.s011.pdf]

On Mar 6, 2023, at 6:03 PM, PLOS Biology <em@editorialmanager.com> wrote:

Dear Dr Lai,

Thank you for your patience while we considered your revised manuscript "Essential and recurrent roles for hairpin RNAs in silencing de novo sex chromosome conflict in Drosophila simulans" for publication as a Research Article at PLOS Biology. Please accept my apologies for the slight delay in getting back to you. Your revised study has been evaluated by the PLOS Biology editors and the Academic Editor who previously provided reviewer comments.

The Academic Editor has evaluated the rebuttal and the revised manuscript and notes that the revision addresses the comments from Reviewer's #1 and #2. However, he/she notes that not all of their concerns have been addressed, such as determining whether the tagged proteins are functional using genetic analyses and clarifying the reporting of the mutant phenotypes. I have provided these specific comments from the Academic Editor below my signature.

In light of these comments, we would like to invite you to revise the work to address these remaining points. Given the extent of revision needed, we cannot make a decision about publication until we have seen the revised manuscript and your response to the Academic Editor's comments.

We expect to receive your revised manuscript within 3 months. Please email us (plosbiology@plos.org) if you have any questions or concerns, or would like to request an extension.

At this stage, your manuscript remains formally under active consideration at our journal; please notify us by email if you do not intend to submit a revision so that we may withdraw it.

IMPORTANT - SUBMITTING YOUR REVISION

Your revisions should address the specific points made by each reviewer. Please submit the following files along with your revised manuscript:

1. A 'Response to Reviewers' file - this should detail your responses to the editorial requests, present a point-by-point response to all of the reviewers' comments, and indicate the changes made to the manuscript.
**NOTE: In your point-by-point response to the reviewers, please provide the full context of each review. Do not selectively quote paragraphs or sentences to reply to. The entire set of reviewer comments should be present in full and each specific point should be responded to individually, point by point.*
You should also cite any additional relevant literature that has been published since the original submission and mention any additional citations in your response.

2. In addition to a clean copy of the manuscript, please also upload a 'track-changes' version of your manuscript that specifies the edits made. This should be uploaded as a "Revised Article with Changes Highlighted" file type.

Re-submission Checklist
When you are ready to resubmit your revised manuscript, please refer to this re-submission checklist:
https://urldefense.com/v3/__https://plos.io/Biology_Checklist__;!!KVWo1iE!UJpdbepyG__q3d-2fn-RhztTMQPhYvT1Ci19Bb6yTny0TkKxbgL8BRoTC5ZkyiWu-wmWx6ilPo1q$

To submit a revised version of your manuscript, please go to https://urldefense.com/v3/__https://www.editorialmanager.com/pbiology/__;!!KVWo1iE!UJpdbepyG__q3d-2fn-RhztTMQPhYvT1Ci19Bb6yTny0TkKxbgL8BRoTC5ZkyiWu-wmWx30b723H$ and log in as an Author. Click the link labelled 'Submissions Needing Revision' where you will find your submission record.

Please make sure to read the following important policies and guidelines while preparing your revision:

Published Peer Review
Please note while forming your response, if your article is accepted, you may have the opportunity to make the peer review history publicly available. The record will include editor decision letters (with reviews) and your responses to reviewer comments. If eligible, we will contact you to opt in or out. Please see here for more details:
https://urldefense.com/v3/__https://blogs.plos.org/plos/2019/05/plos-journals-now-open-for-published-peer-review/__;!!KVWo1iE!UJpdbepyG__q3d-2fn-RhztTMQPhYvT1Ci19Bb6yTny0TkKxbgL8BRoTC5ZkyiWu-wmWx5LDImd3$

PLOS Data Policy
Please note that as a condition of publication PLOS' data policy (https://urldefense.com/v3/__http://journals.plos.org/plosbiology/s/data-availability__;!!KVWo1iE!UJpdbepyG__q3d-2fn-RhztTMQPhYvT1Ci19Bb6yTny0TkKxbgL8BRoTC5ZkyiWu-wmWx0YngoM-$) requires that you make available all data used to draw the conclusions arrived at in your manuscript. If you have not already done so, you must include any data used in your manuscript either in appropriate repositories, within the body of the manuscript, or as supporting information (N.B. this includes any numerical values that were used to generate graphs, histograms etc.). For an example see here:
https://urldefense.com/v3/__http://www.plosbiology.org/article/info*3Adoi*2F10.1371*2Fjournal.pbio.1001908*s5__;JSUlIw!!KVWo1iE!UJpdbepyG__q3d-2fn-RhztTMQPhYvT1Ci19Bb6yTny0TkKxbgL8BRoTC5ZkyiWu-wmWx7NRqDV5$

Blot and Gel Data Policy
We require the original, uncropped and minimally adjusted images supporting all blot and gel results reported in an article's figures or Supporting Information files. We will require these files before a manuscript can be accepted so please prepare them now, if you have not already uploaded them. Please carefully read our guidelines for how to prepare and upload this data:
https://urldefense.com/v3/__https://journals.plos.org/plosbiology/s/figures*loc-blot-and-gel-reporting-requirements__;Iw!!KVWo1iE!UJpdbepyG__q3d-2fn-RhztTMQPhYvT1Ci19Bb6yTny0TkKxbgL8BRoTC5ZkyiWu-wmWx32C3_u9$

Protocols deposition
To enhance the reproducibility of your results, we recommend that if applicable you deposit your laboratory protocols in protocols.io, where a protocol can be assigned its own identifier (DOI) such that it can be cited independently in the future. Additionally, PLOS ONE offers an option for publishing peer-reviewed Lab Protocol articles, which describe protocols hosted on protocols.io. Read more information on sharing protocols at https://urldefense.com/v3/__https://plos.org/protocols?utm_medium=editorial-email&utm_source=authorletters&utm_campaign=protocols__;!!KVWo1iE!UJpdbepyG__q3d-2fn-RhztTMQPhYvT1Ci19Bb6yTny0TkKxbgL8BRoTC5ZkyiWu-wmWx3ccO7J3$

Thank you again for your submission to our journal. We hope that our editorial process has been constructive thus far, and we welcome your feedback at any time. Please don't hesitate to contact us if you have any questions or comments.

Kind regards,

Richard

Richard Hodge, PhD
Associate Editor, PLOS Biology
rhodge@plos.org

ACADEMIC EDITOR COMMENTS

I feel the authors have addressed the comments of reviewers 1 and 2. The figures have improved and the language used is now more precise. However, I do not feel the authors have adequately addressed all of my concerns.

The outstanding major concerns:
1) The authors did not determine that the tagged proteins were functional. Given the strong phenotypes of the tmy and nmy mutants in the presence of functional Dox and Pdox2, this seems quite feasible with genetic analyses. This is also quite important if the authors want to interpret the observed localization.

We recognize the Editor's concern, and provided a full consideration of the issue of how one might "*determine that the tagged proteins were functional*". While this is certainly an interesting and worthwhile experiment, for the many reasons described below, this experimental concern is neither specifically defined by the Editor, nor is "*quite feasible*" in the context of the non-model species D. simulans. Most importantly, it is not critical "*to interpret the observed localization.*" Instead, we are reporting the results from these challenging recombinant genetics experiments from visual selection of unbalanced animals carrying multiple genetic elements, and we describe in full the caveats associated with these results. Nevertheless, we are certain that the readership will be very interested to know these cytological results from Dox and PDox2 transgenes under hpRNA mutant (derepressed) conditions, and they are a springboard for future functional analyses.

Based on the further guidance provided by the Associate Editor "*After discussing with the Academic Editor, we agree that performing the crosses would be outside the scope, but we do ask that any conclusions derived from the constructs are removed in the Abstract and that it is explicitly caveated in the manuscript text that the constructs were not validated.*", we revised as follows.
1. We removed mention from the abstract of the subcellular localization of the derepressed proteins detected in hpRNA mutant conditions, because of the caveat regarding tagging.
2. We do not agree that the abstract should delete "*any conclusions derived from the constructs*". First, we highlight this is the first ever evidence for translated protein products from any Dox family locus. All previous evidence for the past 20 years has been strictly only at the RNA level, or even only as uncloned genetic determinants. Moreover, it was explicitly speculated in the first molecular paper on Dox that it was equally plausible that it might be non-coding vs coding (Tao 2008). Detection of Dox and PDox2 proteins are very germane conclusion from immunostaining. Second, we emphasize their protein products can only be substantially detected under hpRNA mutant conditions, and not in hpRNA proficient genotypes. This is also a very central conclusion to the trans-acting suppression of Dox family loci by Nmy and Tmy hairpin RNAs. We strongly argue these observations are very germane to mention in the abstract.

We hope this major concern has been appropriately discussed and that the text revisions are suitable to the Editor.

Outstanding minor points:
1) The authors did not fully correct the description of the mutant phenotypes, so it was still confusing to know if they were talking about heterozygotes or homozygotes. This applies to the text and figures. Even the example text that i highlighted as being particularly confusing, was not clarified.

We have done our best to clarify the accurate description of genotypes. We consider ourselves to take great care in genetic definitions and nomenclature, in all of our writing. We also spelled out full genotypes, wherever appropriate, the relevant figures to minimize ambiguity. Since the Academic Editor does not specify which particular places they find "*still confusing*", it is challenging to know if we have addressed their concerns appropriately. If they would specifically cite the places of ambiguity, that would aid the discussion. We certainly hope to avoid confusion on this topic, which could be challenging to follow due to the "upside-down" genetic outcomes.

The only thing we can perhaps think is if the Editor was not certain what we mean by "mutant". If this is the case, we note that it is standard genetic parlance that for haplo-sufficient loci (the vast majority of all genes), that "mutant" conditions refer to both alleles being deficient. Thus, where we refer to "*nmy* mutant ", "*tmy* mutant" etc, it is genetic parlance that that refers implicity to both alleles being affected, so we do

not further identify "homozygous". However, in the special cases of haploinsufficiency, or where genetic sensitized backgrounds permit gene heterozygosity to have an overt effect, then it is appropriate to specify heterozygote genotypes. We have done this in the relevant text sections and figures.

We have carefully re-read the text and made additional revisions, which hopefully are suitable.

2) What is now Figure 4C still lacks what to me is a critical control of the tmy/+ genotype at 25 degrees to determine if Tmy is not completely dominant at that temperature. While i appreciate that full characterization of Tmy hets would require testis isolation etc, the authors could assay fertility and the fraction of female progeny with genetic crosses. If such experiments showed no fertility defects or distorted sex ratios, no further experiments would be required.

Our experiments in Fig 4C (now Fig 4D) were aimed at demonstrating genetic enhancement of the nmy mutant sex ratio bias by tmy heterozygosity. These experiments were necessitated because sterility of tmy mutants precluded direct assessment of their impact on progeny sex ratio. We have now included data as the new Fig 4C, which demonstrate that the progeny sex ratios of a cohort of tmy/+ males are indistinguishable from those of the starting w[XD1] recipient strain for CRISPR/Cas9 mutagenesis. We hope these new revision data address the Editor concern.

Further, the authors attribute the phenotype of the tmy/+, nmy/nmy flies to Tmy affecting Dox/MDox drive without sufficient supporting data. The data do not rule out that the phenotype is not due drive of PDox under those experimental conditions.

We agree we cannot fully rule out this possibility, but our conclusions were based on experimental data. (1) We have shown that both Nmy hpRNA and Tmy hpRNA can repress both Dox and MDox in "sufficiency" sensor tests (Lin Dev Cell 2018). (2) We have shown that only Tmy hpRNA, and not Nmy hpRNA, can suppress PDox1/2 in sufficiency sensor tests (this study, Fig 4A). The simplest interpretation consistent with these data is that Dox and MDox might still provide SR drive activity in nmy mutants, which can still be partly suppressed by Tmy in this background. That is a reasonable inference from the experimental tests of which hpRNA can suppress which target (Fig 4F).

We agree it formally remains possible that PDox genes might exert SR drive under these experimental conditions. This would not, however, be very consistent with our observation that tmy/+ does not have SR defect, along with the fact that Nmy is not a suppressor of PDox genes. However, we added an additional caveat to the text to acknowledge it is a formal possibility, and that future tests are needed to fully resolve the functional basis of the interesting genetic interactions we detected between the paralogous Nmy and Tmy hpRNAs, both in male reproductive performance and cytology. We hope this addresses the Academic Editor concern.

New minor points:
Several times, the authors refer to the ages of genes in ways i am not sure are supported by the published data. For example, the natural variants lacking Dox and Pdox2 are described as 'deletions.' Is it clear those genes are not insertions in w[XD1]? Similarly, is it clear that Dox-family-targeting hpRNAs are newly emerged in simulans and were not lost in melanogaster? Is there an out-group whose genome has been assembled well enough to address this question?

Respectfully, it is not typical to raise "*New minor points*" that were not raised by either referees on either previous review. We have clearly shown, in newly revised data, that these Dox family genes are deleted in the wild line X chromosomes, as depicted in schematics and genotyping. It was well-documented in previous papers, as referenced (Vedanayagam Nature Eco Evo 2021 and Muirhead Nature Eco Evo 2021) that these hpRNAs are definitively new-emerged in an ancestor to the simulans clade, as are the Dox family loci. These aforementioned papers report details of the multilocus mobilization of ancestral progenitor loci present in Dmel and other Drosophila species, which led to the presence of current-day PDox/Mdox/Dox family genes in D.simulans and its immediate sister species. The fact that these are "young insertions" within the simulans clade is is not in question, and has been referenced.

Whether one wishes to call it an insertion in w[XD1] relative to NS40, or a deletion in NS40 relative to w[XD1], is slightly semantic. We call them deletions because w[XD1] was the first long-read genome sequenced D. simulans, so it is convenient to reference other genomes relative to w[XD1], at least for the time being. As an analogy, most human genomic variation studies are called in reference to the first available genome(s), out of convenience. Now that there may imminently be 1M human genomes, it is obviously much more complicated to assess what is an insertion or deletion in any given person, its all relative. In any case, it is clear that the ancestral state is for there to be no Dox family loci on the Drosophila X chromosome, and that has been documented; they have inserted recently in an ancestor to the present day D. simulans/sechellia/maurtiana subclade, but also seem to be lost from some present-day D. simulans individuals. Our new data highlight that polymorphic absence of Dox family genes in D.simulans individuals is connected to phenotypic outcomes of hpRNA mutants.

I suggest I suggest the indicated slight changes (in CAPS) to following sentence to avoid the implication that mutations are non-random: "Consequently, the Dox drive system in natural populations likely is often cryptic, and may only manifest following innovationS on the part of the drivers THAT ALLOW THEM TO evade or counter suppression, followed by co-evolutionary responses at Y- linked or autosomal loci.

We edited the text as suggested by the referee.

From: Richard Hodge <rhodge@plos.org>
Subject: [EXTERNAL] RE: Your PLOS Biology Submission (PBIOLOGY-D-22-02436R2)
Date: March 16, 2023 at 6:57:40 PM EDT
To: "Lai, Eric" <laie@mskcc.org>

Dear Eric,

Thank you for your patience here and I am sorry for any confusion that I caused in my decision letter in regard to our editorial process. I should be clear that your manuscript was not sent for re-review by Reviewer #1 and #2. Given the nature of the revisions, we often aim to avoid further rounds of peer review by asking our Academic Editors to directly arbitrate the revision and rebuttal if they are able and have the required expertise. This helps to reduce the burden on reviewer time and often means we can provide authors with a quick decision. In this specific case, our Academic Editor has also acted as a third reviewer for your manuscript, but in the interests of full transparency, we labelled this review as ‘Academic Editor Comments’.

In the previous round, the Academic Editor noted in their review that ‘My first concern is that it isn't clear to me how the authors determined that the fusion proteins were function’. However, we do appreciate that this concern was not made explicit in the review and that no specific experimental suggestion was provided. I have now had a chance to discuss this with the Academic Editor, who had previously assumed that the tagged constructs had been validated in some way and the purpose of this comment was for further clarification on how this had been done. The request to provide genetic crosses to show that the tagged proteins were functional was then requested when it appeared that this validation had not been conducted.

We completely appreciate your arguments that these experiments would be challenging to perform in a non-model organism like Drosophila simulans (with the limited tools available), and may not provide definitive answers anyway. I also understand that our previous decision letter was very encouraging and conveyed the message that only minor experiments, reanalyses and textual changes were envisaged in the revision. After discussing with the Academic Editor, we agree that performing the crosses would be outside the scope, but we do ask that any conclusions derived from the constructs are removed in the Abstract and that it is explicitly caveated in the manuscript text that the constructs were not validated.

I am sorry once again for this and it was my fault in misinterpreting the Academic Editor's comments. We are very much looking forward to publishing your study once these minor textual changes have been made, in addition to the minor comments that were included in our recent decision letter. Please do not hesitate to let me know if you any further questions or concerns.

Best,

Richard

From: Lai, Eric <laie@mskcc.org>
Sent: 16 March 2023 14:28
To: Richard Hodge <rhodge@plos.org>
Subject: Re: Your PLOS Biology Submission (PBIOLOGY-D-22-02436R2)

Dear Richard,
We haven't seen the referee responses.
Since the editor states "I feel the authors have addressed the comments of reviewers 1 and 2",
we are puzzled what else there is to do.
Best,
Eric

****
Eric Lai
Member, Sloan-Kettering Institute
Developmental Biology Program
1017C Rockefeller Research Labs
430 East 67th St. ROC-10, #1013
New York, NY 10065

ph: 212-639-5578
fax: 212-717-3623

https://www.mskcc.org/research-areas/labs/eric-lai

On Mar 15, 2023, at 12:39 PM, Richard Hodge <rhodge@plos.org> wrote:

Dear Eric,

I am very sorry, I did not see your e-mail before I logged off on Monday. I will contact the Academic Editor about your submission today and discuss the decision with the rest of the editorial team. I would just like to reassure you that there is no underlying agenda here in regards to the handling of your manuscript and our intention was not to 'move the goalposts' as it were. We will be back in touch very soon and I am sorry about this once again.

Best wishes,

Richard

From: Lai, Eric <laie@mskcc.org>
Sent: 13 March 2023 20:18

To: Richard Hodge <rhodge@plos.org>
Subject: Re: Your PLOS Biology Submission (PBIOLOGY-D-22-02436R2)

Thank you Richard.
Apologies to bother you on your trip, but we seek confidential clarification on the process.

In our first decision, we were heartened all the referees were very positive, and we were told it would be a minor and quick revision.
We worked hard to fully address the referee concerns on a tight requested schedule, over the Christmas and New years' holidays.

Subsequent to resubmission, we had multiple communcations with PLoS office regarding final formatting and several rounds of author approvals.
Nearly two months later, we were not returned referee responses to our revision.
And now its listed as a major revision with solely Editor-initiated requests, many of which are "new comments".

This seems very irregular handling and that the Editor may have some agenda.
We appreciate your feedback about this, which truly represents some of the work I have been most proud of and most eye-opening from my lab.

Best,
Eric

On Mar 13, 2023, at 6:28 AM, Richard Hodge <rhodge@plos.org> wrote:

Dear Eric,

I am sorry for the delay in getting back to you here, I am currently away on holiday but thought I would just quickly get back to you so you are not kept waiting for me. Thank you for your e-mail in response to the decision we recently sent - I will discuss the arguments you outline with the Academic Editor handling your submission and get back to you with feedback as soon as I can.

Best wishes,

Richard

From: Lai, Eric <laie@mskcc.org>
Sent: 10 March 2023 02:12
To: PLOS Biology <plosbiology@plos.org>; Richard Hodge <rhodge@plos.org>
Subject: Your PLOS Biology Submission (PBIOLOGY-D-22-02436R2)

Dear Richard,
Thanks for returning additional comments.
With the highly positive initial reviews, we made extensive efforts to revise accordingly,
on an initial one month deadline over the holidays ("a revision that we anticipate should not take you very long").

We did not see referee responses to our resubmission, the letter only states "I feel the authors have addressed the comments of reviewers 1 and 2".
Thus we are surprised by new points and experiments raised by the Editor in 2nd review.
Some of these concern additional interpretational caveats and controls.

We revised our text and figures to address all points raised in the original review, but will certainly take further care as requested.

However, we take issue with: "The outstanding major concerns: The authors did not determine that the tagged proteins were functional."
We agree this is an interesting experiment, and could provide useful knowledge.
But this is not central to our main findings, and was not raised by either referee in the first round.
On the contrary, both were enthusiastic about this specific set of difficult tagged transgene experiments we reported.

We respectfully disagree that "this seems quite feasible with genetic analyses".
These are multi-generational crossing schemes that at best would take months just to know an answer.
Even if we technically can draw out how we might do the crosses, finding the individual flies of interest is very challenging in this non-model species.

Most saliently, a potential positive result does not imply anything specific about their location of action, since they appear in multiple compartments.
Conversely, a potential negative result does not distinguish between transgene functionality vs other confounding genetic issues.
For these reasons, both positive and negative outcomes have caveats for what the Editor requests, but actually neither outcome would affect our major conclusions.

The Editor newly implies that acceptance is contingent on some positive rescue test, despite no specific test proposed.
We would not balk if the tests were difficult, but of a defined nature, and would yield definitive conclusions.
But they are neither straightforward, nor easily interpretable, nor critical for our manuscript.
We provide full discussion to enable full editorial consideration of what might seem like a simple test, so we acknowledge this is lengthy.

****

1. Since we analyzed numerous genetic combinations in this study (involving two chomosomes), this might give the impression they were easy tests, but they were not.
Drosophila simulans lacks common genetic tools of D. melanogaster, principally balancer chromosomes, and mostly we cannot make stocks because of ∆tmy sterility.
However, the tests requested require manipulation of three chromosomes.
Just like juggling balls, doing three is substantially more difficult than two.
The animals of interest have to carry a designated wild X chromosome carrying target mutations, and autosomes II and III that carry a transgene and are homozygous for hpRNA mutants.
But, we cannot build stocks to obtain such animals (unlike Dmel), because of no balancers.

2. Further complicating a three-chromosome test is that we cannot visually track all the genetic elements in the proposed animals of interest within unbalanced crosses.
First, we must genotype hpRNA alleles to distinguish heterozygous from homozygous, since this cannot be confidently done by eye.
Second, the transgenes are marked by white+, but this is only visible in the w[XD1] reference genotype.
The wild X genotypes contain a functional white+ gene, so we cannot visually select flies with the transgene from a mixture of progeny.

3. No specific test was proposed by the Editor. In this study, we report two wild X chromosomes that are deficient in different aspects of drive, two hpRNA mutants and two transgenes.
Any individual 3-way combination test is a lot of work and time, and might not succesfully identify the animals of interest (for the reasons above).
But if we need to test each possible combination to have a possibility of rescue, that is 8-fold (2x2x2) of an already long effort. It’s almost a Genetics paper in and of itself.

4. The most important consideration is that the requested tests are not definitive.
Our major conclusion regards that they encode proteins, which are depressed in male germline under meiotic drive conditions.
If any transgene resurrected drive in a wild X; hpRNA mutant, that is of course notable.
But there are many reasons why a negative result would not invalidate transgene functionality.
Its just not interpretable either way with available genotypes.

• 4A. We show in Figure 5 (new data) that the characterized wild suppressor X chromosomes have deletions of multiple dox family loci (MD15: ∆Dox + ∆MDox and NS40: ∆Dox + ∆PDox2).
Thus, it may be necessary to include multiple transgenes in the rescue test, if there are functional collaborations amongst loci (and our data lend plausiblity for this scenario).
It is not practical to control 4 genetic elements in an unbalanced set of crosses. We also do not have an MDox transgene.

• 4B. We did our best to clone relatively large genomic fragments. These transgenes definitively show protein derepression in hpRNA mutants.
However, we cannot rule out if additional transcriptional regulatory elements are needed to achieve endogenous expression needed for drive.
That is not relevant to our conclusion that Dox family genes are functionally and specifically repressed by cognate hpRNAs.
But it does potentially affect the ability for transgenes to resurrect phenotypic drive as discrete DNA fragments, independent of tagging.

• 4C. The drive system loci reside on the X chromosome. Our transgenes happen to be located on autosomes.
If it turns out that some part of the drive activity depends on cognate chromosome location (like XIST), then they may not be fully competent for drive, even though they are clearly de-repressed in hpRNA mutants.
It is not practical to generate all new transgenes from scratch, simply to test this in the context of the multigenerational crossing scheme.

• 4D. The wild X chromosomes may be polymorphic for unknown loci that impact drive.
If this is the case, a functional transgene might not suffice to resurrect drive in a wild X chromosome.
We note Ref 2 understood this issue and specifically applauded our efforts.

"One key contribution of this study is that it allows for a fair comparison between Tmy and Nmy hpRNAs. The authors took great care to generate new mutant alleles to exclude confounding effects due to the random effects caused by introgressions, mutations, and genetic modifiers segregating in populations."

Our experiments show that Dox family target proteins are derepressed in hpRNA knockouts in w[XD1], our reference strain, under phenotypic drive conditions (hpRNA mutants).
Unfortunately, we did not succeed in multiple trials to generate Dox family knockouts in w[XD1], which would be the optimal controlled genetic background to evaluate transgene rescues.
But generating a new panel of individual and combination Dox family knockouts in w[XD1] is surely a future study.
This was already 5+ chronological years and probably 8+ person years to generate and analyze the new hpRNA knockouts and transgenes reported here.

****

The Editor comments "This is also quite important if the authors want to interpret the observed localization",
In this study, we do not claim that Dox family proteins exclusively exert their functions via chromatin, since we clearly described tagged Dox family protein products are not exclusive to nuclei.
We raised alternative mechanistic possibilities in the text, as requested.

Even if the requested tests are positive and transgenes can resurrect drive in a rescue test in wild Xs, we still could not conclude that cytoplasmic or membrane localization of Dox proteins is irrelevant to drive.
It also would not rule out the tag might affect localization but it still happens to work.

Alternatively, the tests may be negative, but in that outcome we still cannot distinguish if the tag affected their function,
or whether tag proteins are functional but reasons of genetic background, necessity for combinations of factors, etc prevent drive resurrection.

Despite the tag caveat, both Reviewers 1 and 2 congratulated our transgene data.
Ref 1: "They further show for the first time that Dox family members code for proteins that are produced only in the absence of their cognate repressors, which are expressed specifically in developing sperm."

Ref 2: "I found the experiments described in figure 4 particularly exciting. Although not the endogenous Dox loci, the visualization of Dox and Dox-like proteins from tagged transgenes in hpRNA mutant background will definitely help characterize the biology of this mysterious protein family."

****

While it is our longterm goal to learn more about these proteins, we do not agree these are critical tests in the context of this manuscript.
This is the first study to report clean, novel hpRNA mutants with profound mutant effects on distinct aspects of spermatogenesis,
with in vivo validation on their endogenous repression of de novo driver loci, and phenotypic connections to sex ratio and sterility, are all fundamental data for the field.
The newly requested, vague, rescue tests will not affect any of these major conclusions, and are multi-month experiments with strong likelihood to not be interpretable.
When I review manuscripts, I will not ask for things if they would significantly delay a study but have no major impact if completed.

We look forward to your feedback and discussion on this issue, so that we can appropriately plan our revision.
We are happy to respond to all the other comments raised.
We hope PLoS Biology will be interested to share this work with the small RNA/RNAi and speciation/evolution communities,
both of which have been very encouraging about the excitement and impact of this work over multiple conference presentations the past two years.

With kind regards,
Eric
